# Multidimensional assessments of impulsivity in women with bulimia nervosa, bipolar disorders, and comorbidity

**DOI:** 10.1186/s40337-025-01319-6

**Published:** 2025-06-17

**Authors:** Bo-Ya Chiu, Mei-Chih Meg Tseng, Yen-Hsiu Liu

**Affiliations:** 1 Department of Primary Care Medicine, Shuang Ho Hospital, Taipei Medical University, New Taipei City, 235041 Taiwan; 2 Department of Psychiatry, School of Medicine, College of Medicine, Taipei Medical University, Taipei, 100301 Taiwan; 3 Department of Psychiatry, Shuang Ho Hospital, Taipei Medical University, New Taipei City, 235041 Taiwan; 4 Department of Psychiatry, National Taiwan University College of Medicine, Taipei, 100233 Taiwan; 5 Department of Psychiatry, Shuang Ho Hospital, No. 291, Zhongzheng Rd., Zhonghe District, New Taipei City, 235041 Taiwan

**Keywords:** Affect lability, Bipolar disorder, Bulimia nervosa, Comorbidity, Impulsivity, Negative urgency

## Abstract

**Background:**

This study investigated the shared and distinct features of emotional dysregulation and impulsivity in women with bulimia nervosa (BN) and bipolar disorder (BD) as well as their relationship with comorbidity between the two conditions.

**Method:**

This study included data from 115 women with BN and 76 women with BD, including 37 individuals with comorbid BN–BD, from a psychiatric outpatient clinic as well as 72 healthy female controls. All participants underwent a diagnostic interview and completed several self-administered assessments of mood and impulsivity. Statistical analyses were conducted to compare mood and impulsivity across the BN-only, BD-only, BN–BD comorbid, and control groups.

**Results:**

The disorder groups exhibited significantly higher levels of affective lability, attentional impulsivity, sensitivity to punishment, negative urgency, and both internally and externally directed impulsive behaviors compared with the control group. Comorbidity with BN–BD was further associated with increased severity in anger, attentional impulsivity, reward sensitivity (fun seeking), and externally directed impulsive behaviors relative to the BN-only group. Among these shared psychopathological features, negative urgency was significantly higher in the BN-only and BN–BD comorbid groups than in the BD-only group, indicating its unique relevance to binge eating. Attentional impulsivity was the only parameter that demonstrated significantly greater severity in the comorbidity group than in both the BN-only and BD-only groups. This finding implies that attentional impulsivity is associated with an elevated risk of externally directed impulsive behaviors in the BN–BD comorbid group relative to the BN-only group.

**Conclusions:**

BN and BD share overlapping affect dysregulation and impulsivity profiles. Negative urgency and attentional impulsivity may make major contributions to binge eating and external impulsive behaviors, respectively, in individuals with comorbid BN–BD. Our findings highlight the importance of targeting emotion regulation skills as well as behavioral control of binge eating and associated impulsive behaviors when treating individuals with BN and BD comorbidity.

## Abstract (plain language summary)

This study explores how emotional ups and downs and impulsivity are similar and different in women with bulimia nervosa (BN) and bipolar disorder (BD). It looked at 115 women with BN, 76 with BD, 37 individuals with comorbid BN–BD, and 72 female controls. The researchers conducted interviews and used questionnaires to assess mood and impulsivity. They found that individuals with BN and BD showed much higher levels of emotional instability and impulsivity compared to healthy individuals, and the comorbid group (BN–BD) showed significantly greater severity of externally directed impulsive behaviors than those with BN-only. Specifically, the tendencies to eat an enormous amount of food in response to negative emotions are of particular relevance to binge eating in people with BN and those with both BN and BD and having difficulty in maintaining focus or resisting distraction may be related to the additional impulsive behaviors in people with BN-BD. The study emphasizes the need to target emotion regulation skills as well as behavioral control of binge eating and associated impulsive behaviors when assisting individuals facing both BN and BD.

## Introduction

Bipolar disorder (BD) is characterized by alternating episodes of depression and mania or hypomania, often accompanied by impaired judgment, heightened activity, and impulsive behavior [1]. Eating disorders (EDs) encompass a range of conditions involving abnormal eating habits and distorted body image, including anorexia nervosa, bulimia nervosa (BN), and binge-eating disorder (BED) [2]. Both BD and ED share core phenomenological features, including mood disturbance, dysregulated eating, impulsivity, and weight problems [3]. The comorbidity of ED in BD is associated with a more severe negative illness course and outcome, and increased risk of additional clinical comorbidities, including suicidality and alcohol and substance use disorders among in the BD population [3, 4]. ED patients with comorbid BD also exhibit increased risks of weight dysregulation, suicidality, impulsivity, and higher rates of psychiatric comorbidities within the ED population [5]. Among EDs, BN and BED have the highest prevalence in individuals with BD [3]. BN is characterized by recurrent episodes of binge eating followed by inappropriate compensatory behaviors, such as vomiting, fasting, or excessive exercise, and frequently associated with elevated food-related impulsivity [6, 7]. Negative affect often exists in BN and emotional dysregulation has been proposed to increase the risk of binge eating [8]. These converging findings underscore that impulsivity and poor emotion regulation are key characteristics tied to binge-eating pathology in BN. Theoretical models have hypothesized that BD and ED are separate but related pathophysiological manifestations involving dysregulation in mood, eating behavior, body weight, and impulse control, as evidenced by a substantial overlap between these disorders in terms of phenomenology, course, comorbidity, family history, and response to pharmacological treatment [9]. Although emotion dysregulation and impulsivity have been studied in BD [10, 11] and ED respectively; the impulsivity parameters in relation to ED-BD comorbidity are still relatively unknown, specifically the relationship between impulsivity and ED–BD comorbidity among ED populations [5, 12]. This leaves the knowledge gap regarding whether different facets of impulsivity differ in their contribution to binge eating and additional impulsive behaviors in ED-BD.

Impulsivity is a predisposition toward rapid, unplanned reactions to internal or external stimuli, with no regard for the negative consequences of such actions on oneself or others [13]. It is a complex and multidimensional construct that encompasses several interrelated domains and has been extensively studied using various psychometric tools. Among the various models linking predisposition to impulsivity, negative urgency—the tendency to act impulsively during distress—has been particularly predictive of binge-eating behavior [14, 15]. This has been supported by findings from behavioral impulsivity tasks (e.g., go/no-go) [16] and elevated scores on the UPPS scale [17]. Developed on the basis of a factor analysis of multiple recognized impulsivity measures, the UPPS model involves four constructs: lack of planning (i.e., the tendency to engage in behaviors with limited advance planning), lack of perseverance (i.e., limited capacity for maintaining focus in the face of distraction), sensation seeking, and urgency (i.e., to engage in rash action in response to intense negative emotions) [18]. A meta-analysis reported that patients with BD have significantly higher negative urgency scores, with large effect sizes, than controls do and that emotional urgency is a stable trait of BD, regardless of illness phases or mood state [19]. Three constructs, including attentional impulsivity (difficulty maintaining focus or resisting distraction), motor impulsivity (acting without thinking), and nonplanning impulsivity (a tendency to neglect future consequences in decision-making) [20], assessed by the Barratt Impulsiveness Scale (BIS-11), have been linked to maladaptive behaviors, including binge eating and impulsive behaviors. Studies have consistently demonstrated that patients with BN have higher scores than controls, particularly on the BIS-11, particularly on the attentional impulsivity subscale [21]. However, findings regarding motor and nonplanning impulsivity have been mixed [22–29]. Similarly, in individuals with BD, impulsivity—as measured by the BIS-11 and its subscales—is elevated compared to healthy controls across all illness phases [30, 31]. In parallel, reward and punishment sensitivity—conceptualized through Gray’s reinforcement sensitivity theory—represent motivational systems that drive behavior toward pleasurable outcomes or away from aversive stimuli. These tendencies are typically assessed using the Behavioral Inhibition System/Behavioral Activation System (BIS/BAS) scale, which measures an individual’s responsiveness to reward (BAS) and punishment cues (BIS) [32, 33]. A meta-analysis of the BIA/BAS scales in patients with clinical ED, along with two subsequent studies, revealed that patients with BN are more sensitive to punishment than healthy controls, whereas reward sensitivity was not consistently higher in patients with BN than in controls [34–36]. Few studies, however, have examined BIS/BAS functioning in patients with BD.

The current study aimed to investigate shared and unique dimensions of impulsivity in women with BN and BD using multifaceted assessment tools; specifically, to examine differences in impulsivity parameters between the comorbid BN-BD and disease-only groups. We hypothesize that negative urgency plays a central role in binge eating, such that individuals with BN—regardless of comorbid BD—will show higher UPPS-urgency scores than those with BD alone, and both clinical groups will score higher than controls. We further expect that certain impulsivity dimensions will be elevated in the comorbid BN-BD group compared to both disease-only groups, and that these elevations may contribute to the additional impulsive behaviors observed in BN-BD. Clarifying the distinct and common impulsivity profiles may enhance our understanding of underlying mechanisms, improve diagnostic precision, and inform tailored interventions for individuals with comorbid presentations.

## Methods

### Participants and procedures

The data of patients with BN and healthy controls were recruited from a previous study [5]. In that study, patients with EDs were enrolled consecutively from the general outpatient clinics of the Department of Psychiatry at a teaching hospital using a two-phase method. Psychiatric outpatients who consented to participate completed a brief paper-form screening questionnaire, the SCOFF [37]. Those who met the screening criteria were subsequently interviewed using the ED Module of the Structured Clinical Interview for DSM-IV-TR Axis I Disorders, Patient version (SCID-I/P) [38]. The healthy controls, matched to BN patients in age, gender, and education level, were recruited through public advertisements. Due to the small number of male BN participants (*n* = 5), we restricted our analysis to female patients to ensure sample homogeneity. During the same period, a group of female patients with BD was consecutively recruited from the same psychiatric clinics. The exclusion criteria for the study included age below 18 or above 45 years, active psychotic conditions, organic mental disorders, mental disability, and severe physical conditions. All the participants completed the Mini-International Neuropsychiatric Interview (MINI) [39] to confirm their psychiatric diagnoses and completed various questionnaires evaluating eating pathology, mood, and impulsivity. The control participants were screened to ensure that they had no history of ED, no current major psychiatric or medical conditions, no use of psychoactive medications, and no drug or alcohol abuse. The study protocol was approved by the Institutional Review Board of National Taiwan University Hospital.

### Diagnostic interview

Axis I diagnoses for patients with BD and BN were determined using the MINI [39] and the ED module of the SCID-I/P [38], respectively. These diagnostic tools were adapted to align with the *Diagnostic and Statistical Manual of Mental Disorders*,* Fifth Edition* (*DSM-5*) criteria. All interviews were conducted by a psychiatrist and two research assistants, both of whom had undergraduate degrees in psychology. The interrater reliability, measured using κ coefficients, was 0.82 for BD and 1.00 for BN.

### Measures

#### Barratt impulsiveness scale (BIS-11)

The BIS-11 is a 30-item self-report questionnaire designed to measure trait impulsivity [20]. The Chinese version of the BIS-11 (BIS-11-CH) was adapted from the original by removing five items (items 15, 21, 23, 27, and 29) that exhibited poor item-total correlations (< 0.1) [40]. The resulting 25-item BIS-11-CH has a Cronbach’s α of 0.83 and an intact factorial structure comprising factors related to an inability to plan, lack of perseverance and self-control, and propensity toward novelty seeking and acting without thinking [40]. These three factors represent similar psychological constructs to the following order as those originally identified [20]: nonplanning impulsiveness, attentional impulsiveness, and motor impulsiveness.

#### Behavioral Inhibition system/behavioral activation system (BIS/BAS) scales

The BIS/BAS scales are designed to assess two fundamental motivational systems: the BIS reflects an individual’s sensitivity to punishment, aversive stimuli, and conflict, and is associated with anxiety and behavioral avoidance; whereas the BAS reflects sensitivity to reward and is associated with goal-directed behavior, positive affect, and impulsivity. Responsiveness to reward was further divided into three subconstructs: positive mood and energy in response to achieving or anticipating a desired outcome, assessed by the BAS-reward responsiveness subscale (BAS-RR), effortful and energetic pursuit of goals, assessed by the BAS-drive subscale (BAS-D), and impulsive pursuit of pleasurable opportunities, assessed by the BAS-fun seeking subscale (BAS-FS). It is a 20-item instrument that uses a 4-point Likert-type scale, where 1 indicates strong agreement and 4 indicates strong disagreement. The Chinese version of the BIS/BAS exhibits robust psychometric properties [41]. The internal consistency, as measured using Cronbach’s α, is 0.75 for BIS and in the range of 0.75−0.85 for BAS. The test–retest reliability for the BIS and BAS subscales is 0.72 and ranges from 0.44−0.67, respectively, with the BAS-RR scale exhibiting the lowest test–retest reliability.

#### UPPS, urgency subscale (UPPS-urgency)

The UPPS Impulsive Behavior Scale is a 4-point Likert-type scale, with endpoints ranging from 1 (*strongly agree*) to 4 (*strongly disagree*). It assesses four impulsivity dimensions: lack of planning, lack of perseverance, sensation seeking, and urgency. Urgency refers to the tendency to act impulsively in response to intense emotional states, particularly negative emotions such as distress, frustration, or anxiety. In this study, we used the 12-item urgency subscale. The internal consistency of the Chinese version of the UPPS-urgency is strong (Cronbach’s α = 0.88), and the item-total correlations for this subscale are generally satisfactory.

#### Impulsive behavior scale (IBS)

The IBS is a self-report instrument that evaluates the extent to which individuals engage in 25 impulsive behaviors [42]. Respondents rate the frequency of each behavior on a 5-point Likert-type scale (ranging from 1 = *never*, 2 = *once*, 3 = *on occasion [two to three times in your life]*, 4 = *sometimes [4–20 times in your life]*, and 5 = *regularly [more than 20 times in your life]*). The items are categorized under two subscales: internally directed impulsive behaviors (11 items) and externally directed impulsive behaviors (14 items) [43]. The IBS has been validated in Chinese populations [5], and the scale has robust internal consistency (Cronbach’s α = 0.87).

#### Mood and eating-related measures

State-Trait Anxiety Inventory (STAI). The STAI is a self-report instrument using a 4-point Likert scale and comprises 40 items, including 20 items assessing state anxiety and 20 measuring trait anxiety [44]. The state anxiety items measure individuals’ temporal feelings of fear or worry, whereas the trait anxiety items measure the tendency to experience anxiety. In this study, the Chinese version of the STAI demonstrated strong reliability, with Cronbach’s α values of 0.93 and 0.91 for state and trait anxiety, respectively.

Beck Depression Inventory. The Beck Depression Inventory (BDI) is a 21-item self-report instrument designed to assess the severity of depressive symptoms experienced over the past week. The Chinese version of the BDI exhibits strong reliability and construct validity [45].

Affect Lability Scale. The Affect Lability Scale (ALS) is a 54-item self-report measure that assesses individuals’ tendencies to experience mood fluctuations, capturing shifts between their perceived normal mood and the affective states of anger, depression, elation, and anxiety. It also evaluates individuals’ tendency to oscillate between depression and elation as well as between depression and anxiety [46]. Oliver and Simons developed an 18-item short form of the scale (ALS-18) [47]. The Chinese version of the ALS-18 has demonstrated excellent internal consistency, with a Cronbach’s α of 0.93.

Bulimic Investigatory Test Edinburgh. The Bulimic Investigatory Test Edinburgh (BITE) is a self-report instrument for identifying symptoms of binge eating and assessing severity and treatment response. This 36-item self-reported measure includes two subscales: the Symptom Scale (30 items) and the Severity Scale (six items) [48]. The Symptom Scale uses “yes” or “no” responses, while the Severity Scale is scored based on the frequency of binge eating or purging behaviors. The Chinese version of the BITE exhibits strong test–retest reliability, with intraclass correlation coefficients of 0.86 and 0.88 for the two subscales, respectively [49].

Body Shape Questionnaire. The Body Shape Questionnaire (BSQ) was originally designed to assess body shape concerns and their emotional and behavioral impact. In this study, the BSQ-8 derived from the Chinese version of the BSQ [50], was used. The BSQ-8 has demonstrated favorable internal consistency, with a Cronbach’s α of 0.88.

### Statistical analysis

We compared patients with BN or BD to healthy controls. Descriptive statistics were computed to determine means and standard deviations for all continuous variables and frequency distributions for categorical variables. For categorical variables, frequency differences between groups (BN vs. controls; BD vs. controls) were compared using the chi-square test or Fisher’s exact test. Subsequently, homogeneity and normality of variances were tested, after which Student’s *t* test was performed to evaluate the differences between patients with BN or BD and controls. Effect sizes for pairwise comparisons were measured using Cohen’s d coefficient, interpreted as low (d > 0.2), moderate (d > 0.5), and large (d > 0.8; [51]. To examine the effect of comorbidity on emotional regulation and impulsivity, we regrouped the participants into the following four groups: BN-only, BN–BD comorbid, BD-only, and control groups. Differences in continuous variables between these groups were compared using one-way analysis of variance (ANOVA), followed by Tukey’s test for post hoc comparisons. All tests were two-tailed, and *P* <.05 was considered significant. All statistical analyses were conducted using SPSS 18.0 (SPSS Inc., Chicago, IL, USA) for Windows.

## Results

### Demographics and clinical characteristics

In total, 115 women met the *DSM-5* criteria for BN and 76 for BD. Among these, 78 were given a diagnosis of BN-only, 39 of BD-only, and 37 of comorbid BN–BD. Among the patients with BD, 68 (94.4%) were in a depressive episode, and 8 were in a euthymic state. Additionally, 72 healthy female controls were included in the study. No significant differences in age, education levels, marital status, or living arrangements were noted between the disorder groups (BN and BD groups) and the control group. The mean age of BN and BD onset was 18.5 years and 20.7 years, respectively. While current and maximal body mass index (BMI) did not significantly differ across groups, the minimal BMI was significantly lower in both the BN and BD groups compared to controls (Table 1).

**Table 1:** Basic and clinical characteristics of the participants, categorized by clinical diagnosis

Characteristic	Bulimia nervosa (BN)	Bipolar disorder (BD)	Control group (C)	BN vs. C *P*	BD vs. C *P*
	(*n* = 115)	(*n* = 76)	(*n* = 72)		
Age (years)	25.9 ± 6.8	27.1 ± 7.4	26.4 ± 7.0	0.670	0.524
Education (years)	14.5 ± 2.6	14.5 ± 3.2	15.1 ± 2.4	0.079	0.205
Highest parental education (years)	13.1 ± 4.2	13.1 ± 4.4	12.8 ± 4.1	0.683	0.724
BMI (kg/m^2^)*					
Current	21.1 ± 2.7	21.8 ± 3.8	21.6 ± 3.4	0.268	0.719
Maximal	23.9 ± 4.0	24.4 ± 4.8	23.0 ± 3.5	0.123	0.054
Minimal	17.7 ± 2.2	18.1 ± 2.4	19.6 ± 2.5	< 0.001	< 0.001
Married	17 (14.8%)	12 (15.8%)	6 (8.3%)	0.191	0.165
Living alone	13 (11.3%)	8 (10.5%)	5 (6.9%)	0.325	0.442

Values indicate N (%) or mean ± SD; *P* indicates statistical value by Student *t* test

*Note. Minimum and maximum BMI indicate body weight index after participants reached 18 years of age

### Comparisons of mood symptoms between BN and BD and control groups

As depicted in Table 2, the participants in the disorder groups scored significantly higher on the BSQ and BITE compared with the control group. Similarly, anxiety, depression, and affective lability, as assessed using the STAI, STAS, BDI, and ALS, were significantly elevated in the disorder groups compared with the control group.

**Table 2:** Comparison of eating-specific and general psychological measures between BN and BD and control groups

Measure	Bulimia nervosa (BN)	Bipolar disorder (BD)	Control group (C)	BN vs. C *P*	BD vs. C *P*
	(*n* = 115)	(*n* = 76)	(*n* = 72)		
	Mean ± SD	Mean ± SD	Mean ± SD		
Body Shape Questionnaire	5.0 ± 1.0	4.0 ± 1.4	2.5 ± 1.0	< 0.001	< 0.001
Bulimic Investigatory Test Edinburgh	36.6 ± 8.6	23.8 ± 14.6	4.7 ± 3.8	< 0.001	< 0.001
State-Trait Anxiety Inventory, State	54.3 ± 12.7	54.4 ± 13.3	35.1 ± 6.9	< 0.001	< 0.001
State-Trait Anxiety Inventory, Trait	58.8 ± 9.8	58.4 ± 10.0	41.0 ± 8.2	< 0.001	< 0.001
Beck Depression Inventory	24.7 ± 10.6	25.6 ± 11.9	5.7 ± 6.4	< 0.001	< 0.001
Affective Lability Scale	50.1 ± 10.2	51.3 ± 10.9	32.7 ± 9.5	< 0.001	< 0.001
Anxiety/depression subscale	15.7 ± 3.4	15.6 ± 3.6	8.9 ± 3.0	< 0.001	< 0.001
Depression/elevation subscale	21.6 ± 4.6	22.0 ± 4.7	15.7 ± 4.8	< 0.001	< 0.001
Anger subscale	12.9 ± 3.7	13.7 ± 4.0	8.1 ± 2.7	< 0.001	< 0.001

*P* indicates statistical value by Student *t* test

### Comparisons of impulsivity between BN and BD and control groups

The disorder groups scored significantly higher than the control group on the BIS-11-CH total, BIS-1-CH-lack of self-control, BIS-11-CH-inability to plan, BIS/BAS total, BIS, UPPS-urgency, and IBS (total, internally directed impulsivity, and externally directed impulsivity), with effect sizes ranging from medium to large (Table 3). No significant differences were observed on the BIS-11-CH novelty seeking and BAS subscales between the disorder groups and controls. The only exception was that the BN group scored lower than the control group on the BAS-D subscale, although the effect size was small.

**Table 3:** Comparison of impulsivity-related psychological measures between BN and BD and control groups

Measure	Bulimia nervosa (BN)	Bipolar disorder (BD)	Control group (C)	BN vs. C *P*	BD vs. C *P*	BN vs. C Effect size	BD vs. C Effect size
	(*n* = 115)	(*n* = 76)	(*n* = 72)				
	Mean ± SD	Mean ± SD	Mean ± SD			Cohen’s d	Cohen’s d
BIS-11-CH	60.8 ± 10.9	62.1 ± 11.8	53.9 ± 8.2	< 0.001	< 0.001	0.69	0.80
BIS-11-CH-lack of self-control	27.5 ± 5.5	28.0 ± 6.2	23.0 ± 4.3	< 0.001	< 0.001	0.89	0.93
BIS-11-CH-novelty seeking	13.6 ± 2.9	14.3 ± 3.0	13.6 ± 2.5	0.982	0.106	0.00	0.27
BIS-11-CH-inability to plan	19.8 ± 4.7	19.8 ± 4.7	17.4 ± 4.0	0.001	0.001	0.53	0.55
BIS/BAS	75.8 ± 7.1	75.8 ± 7.2	72.6 ± 5.7	0.001	0.004	0.49	0.49
BIS	23.7 ± 3.2	23.0 ± 3.1	20.2 ± 2.7	< 0.001	< 0.001	1.16	0.97
BAS-drive	12.0 ± 2.3	12.3 ± 2.2	12.8 ± 1.9	0.021	0.165	−0.35	−0.23
BAS-fun seeking	11.3 ± 2.3	12.0 ± 2.3	11.5 ± 1.8	0.569	0.149	−0.09	0.24
BAS-reward responsiveness	17.2 ± 2.2	17.0 ± 2.2	17.4 ± 2.0	0.393	0.263	−0.13	−0.18
UPPS-urgency	37.4 ± 6.4	36.1 ± 7.6	24.7 ± 6.9	< 0.001	< 0.001	1.93	1.57
Impulsive Behavior Scale	49.0 ± 14.1	53.4 ± 16.2	31.3 ± 5.7	< 0.001	< 0.001	1.52	1.80
Internally directed	24.6 ± 8.2	26.5 ± 9.0	13.3 ± 2.8	< 0.001	< 0.001	1.68	1.95
Externally directed	24.4 ± 7.6	26.9 ± 8.8	17.9 ± 3.7	< 0.001	< 0.001	1.01	1.30

BIS-11-CH: Chinese version of Barratt Impulsiveness Scale; BIS/BAS: Behavioral Inhibition System/Behavioral Activation System

### Comparisons of mood and impulsivity across comorbid, BN-only, BD-only, and control groups

When the disorder groups were divided into comorbid BN-BD and BN- and BD-only groups, notable differences were observed (Table 4). There were no significant differences among the comorbid, BN-only, and BD-only groups on STAI, STAS, or BDI. However, both disorder groups scored significantly higher than the controls did on these measures. The comorbid group showed significantly higher scores on the ALS total and the anger subscale compared to the BN-only group and controls. However, there was no significant difference between the comorbid and BD-only groups for these measures.

**Table 4:** Comparison of eating and mood symptoms across comorbid, BN-only, BD-only, and control groups

Measure	Bulimia nervosa only (1)	Bulimia nervosa with bipolar disorder (2)	Bipolar disorders only (3)	Control group (4)	*P*	Post hoc comparison
	(*n* = 78)	(*n* = 37)	(*n* = 39)	(*n* = 72)		
	Mean ± SD	Mean ± SD	Mean ± SD	Mean ± SD		
Body Shape Questionnaire	5.0 ± 1.1_a_	5.0 ± 1.0_a_	3.2 ± 1.2_b_	2.5 ± 1.0_b_	< 0.001	1, 2 > 3 > 4
Bulimic Investigatory Test Edinburgh	36.8 ± 8.5_a_	36.1 ± 8.9_a_	12.2 ± 7.8_b_	4.7 ± 3.8_c_	< 0.001	1, 2 > 3 > 4
State-Trait Anxiety Inventory, State	52.7 ± 12.6_a_	57.7 ± 12.5_a_	51.2 ± 13.4_a_	35.1 ± 6.9_b_	< 0.001	1, 2, 3 > 4
State-Trait Anxiety Inventory, Trait	58.1 ± 10.4_a_	60.5 ± 8.2_a_	56.4 ± 11.2_a_	41.0 ± 8.2_b_	< 0.001	1, 2, 3 > 4
Beck Depression Inventory	23.4 ± 10.9_a_	27.3 ± 9.4_a_	24.1 ± 13.7_a_	5.7 ± 6.4_b_	< 0.001	1, 2, 3 > 4
Affective Lability Scale	48.4 ± 10.1_a_	53.7 ± 9.5_b_	49.0 ± 11.8_ab_	32.7 ± 9.5_c_	< 0.001	2 > 1 > 4; 3 > 4
Anxiety/depression subscale	15.3 ± 3.5_a_	16.5 ± 3.2_a_	14.8 ± 3.8_a_	8.9 ± 3.0_b_	< 0.001	1, 2, 3 > 4
Depression/elevation subscale	20.9 ± 4.8_a_	22.9 ± 4.0_a_	21.2 ± 5.2_a_	15.7 ± 4.8_b_	< 0.001	1, 2, 3 > 4
Anger subscale	12.2 ± 3.6_a_	14.4 ± 3.6_b_	13.0 ± 4.2_ab_	8.1 ± 2.7_c_	< 0.001	2 > 1 > 4; 3 > 4

Means with different subscripts differ significantly at α = 0.05, as indicated by Tukey’s Honestly Significant Difference test

Regarding the impulsivity measures presented in Table 5, the BN-only group scored significantly higher than controls on the BIS-11-CH total, BIS-11-CH lack of self-control, BIS-11-CH inability to plan, BIS, UPPS urgency, and IBS (total, internally directed, and externally directed behaviors) (1 > 4). The BD-only group also scored significantly higher than controls on the BIS-11-CH total, BIS-11-CH lack of self-control, BIS, UPPS urgency, and IBS (total, internally directed, and externally directed behaviors) (3 > 4). The comorbid group had significantly higher scores than the BN-only group on the BIS-11-CH total, BIS-11-CH lack of self-control, BAS-FS, and IBS total and externally directed behaviors (2 > 1). Additionally, the comorbid group had significantly higher scores on the BIS-11-CH-lack of self-control, BIS/BAS total, and BAS-RR than the BD-only group did (2 > 3). Finally, the BN-only group scored significantly higher than the BD-only group on the UPPS urgency subscale.

**Table 5:** Comparison of impulsivity-related psychological measures across comorbid, BN-only, BD-only, and control groups

Measure	Bulimia nervosa only (1)	Bulimia nervosa with bipolar disorder (2)	Bipolar disorders only (3)	Control group (4)	*P*	Post hoc comparison
	(*n* = 78)	(*n* = 37)	(*n* = 39)	(*n* = 72)		
	Mean ± SD	Mean ± SD	Mean ± SD	Mean ± SD		
BIS-11-CH	59.0 ± 9.6_a_	64.5 ± 12.6_b_	59.8 ± 10.6_ab_	53.9 ± 8.2_c_	< 0.001	2 > 1 > 4; 3 > 4
BIS11CH-lack of self-control	26.3 ± 4.6_a_	30.0 ± 6.4_b_	26.1 ± 5.3_a_	23.0 ± 4.3_c_	< 0.001	2 > 1, 3 > 4
BIS11CH-novelty seeking	13.1 ± 2.6_a_	14.5 ± 3.3_a_	14.2 ± 2.8_a_	13.6 ± 2.5_a_	0.066	–
BIS11CH-inability to plan	19.6 ± 4.6_a_	20.1 ± 4.8_a_	19.6 ± 4.7_ab_	17.4 ± 4.0_b_	0.005	1, 2 > 4
BIS/BAS	74.6 ± 7.2_ab_	78.2 ± 6.3_a_	73.7 ± 7.4_b_	72.6 ± 5.7_b_	0.001	2 > 3, 4
BIS	23.7 ± 3.2_a_	23.7 ± 3.4_a_	22.4 ± 2.8_a_	20.2 ± 2.7_b_	< 0.001	1, 2, 3 > 4
BAS-drive	11.9 ± 2.4_a_	12.3 ± 2.2_a_	12.3 ± 2.2_a_	12.8 ± 1.9_a_	0.093	–
BAS-fun seeking	10.8 ± 2.1_a_	12.5 ± 2.1_b_	11.6 ± 2.5_ab_	11.5 ± 1.8 _ab_	0.001	2 > 1
BAS-reward responsiveness	16.9 ± 2.2_ab_	17.8 ± 2.2_a_	16.3 ± 2.0_b_	17.4 ± 2.0_a_	0.008	2, 4 > 3
UPPS-urgency	36.4 ± 6.4_a_	39.5 ± 5.8_a_	32.9 ± 7.7_b_	24.7 ± 6.9 _c_	< 0.001	1, 2 > 3 > 4
Impulsive Behavior Scale	46.1 ± 11.3_a_	55.1 ± 17.4_b_	51.7 ± 15.1_ab_	31.3 ± 5.7 _c_	< 0.001	2 > 1 > 4; 3 > 4
Internally directed	23.5 ± 7.5_a_	26.9 ± 9.3_a_	26.1 ± 8.8_a_	13.3 ± 2.8 _b_	< 0.001	1, 2, 3 > 4
Externally directed	22.6 ± 5.5_a_	28.2 ± 9.8_b_	25.6 ± 7.7_ab_	17.9 ± 3.7 _c_	< 0.001	2 > 1 > 4; 3 > 4

BIS-11-CH: Chinese version of Barratt Impulsiveness Scale; BIS/BAS: Behavioral Inhibition System/Behavioral Activation System;

Means with different subscripts differ significantly at α = 0.05, as indicated by Tukey’s Honestly Significant Difference test

## Discussion

Our results indicate that both the BN and the BD group exhibited significantly higher levels of emotional distress and impulsivity across most measures than the control group did, with a few exceptions—specifically on the BIS-11-CH novelty seeking subscale and most BAS subscales. These results align with prior research suggesting that impulsivity-related traits—especially those involving attentional impulsivity and emotion-driven action tendencies—are relevant across both BN and BD populations. The presence of BN–BD comorbidity, however, was associated with increased severity in anger, attentional impulsivity, reward sensitivity (fun seeking), and externally directed impulsive behaviors relative to people of the BN-only group. The BN–BD comorbid group exhibited greater severity in attentional impulsivity, sensitivity to reward (reward responsiveness), and negative urgency than the BD-only group. The BN-only and BD-only group also displayed higher levels of attentional impulsivity, sensitivity to punishment, and negative urgency than controls, indicating they were shared by the disease-only groups despite the absence of comorbidity. Moreover, the differential degree of severity in three impulsivity constructs across the disease-only, the comorbid, and control groups may have reflect distinct disorder-specific traits as well as additive or synergistic effects in the comorbid group.

In alignment with our earlier findings that patients with ED with comorbid BD had significantly higher risks of suicidality, impulsive behaviors, and psychiatric comorbidities [5], the present study demonstrated that BN–BD comorbidity was associated with a significantly greater severity of externally directed impulsive behaviors, such as reckless driving, alcohol use, sexual promiscuity, stealing, and excessive buying, than the BN-only group was. Notably, attentional impulsivity—as measured by the BIS-11-CH lack of self-control subscale— was the only parameter that exhibited significantly increased severity in the comorbidity group compared with both the BN-only and BD-only groups. This finding suggests that attentional impulsivity may be an underlying mechanism associated with a greater severity of externally-directed behaviors in BN-BD comorbidity beyond binge eating. This interpretation is supported by the finding that previous history of suicide attempts was specifically associated with attentional impulsivity (not with motor impulsivity and nonplanning impulsivity) [52]. Attentional impulsivity has been consistently associated with overeating [21]; in BN, this trait may underlie the difficulty resisting urges in response to emotional or environmental (food-related) cues—consistent with the subjective sense of loss of control during binge eating. Patients with BN–BD exhibited greater attentional impulsivity than those with BN-only, but the two groups showed comparable levels of binge-eating severity. This suggests that while BD comorbidity may amplify attentional impulsivity, it does not appear to exacerbate the loss of control associated with binge eating in individuals with BN.

While findings on nonplanning impulsivity have been inconsistent—with some studies showing higher scores in BN [27, 28] and others showing no group differences [23, 29]. Our study supports it may still reflect a tendency toward poor behavioral regulation that contributes to binge eating. As for motor impulsivity, our findings showed no group differences, which is consistent with two of the four prior studies [22, 23, 27, 29], suggesting that this facet may be less relevant to the impulsivity profile of BN. The literature findings regarding the BIS-11 subscales in BD are complex because of the disorder’s different mood phases. Research has indicated that the three components of impulsivity, as measured using the BIS-11, were related differently to different affective states in BD [22, 53]. Specifically, motor impulsivity was reported to be correlated with mania scores, whereas nonplanning impulsivity was reported to be correlated with depression scores. Additionally, total and attentional impulsivity is independently correlated with depression and mania scores. In our study, most patients with BD were in a depressive state, which may account for the lack of significant differences in motor impulsivity (measured by the BIS-11-novelty seeking) between the patients with BD and controls.

In our sample, individuals with BN and BD exhibited higher punishment sensitivity, a finding that aligns with prior literature [54, 55]. Moreover, the severity of punishment sensitivity was comparable across the BN-only, BD-only, and comorbid BN-BD groups, suggesting that this trait may be a general vulnerability factor rather than one specifically linked to binge eating or additional impulsive behaviors in the comorbid group. In contrast, findings regarding reward sensitivity have been less consistent. Three studies [22, 34, 36] reported no differences in BAS subscale scores between the BN and control groups, and only one study reported higher BAS-RR scores in patients with BN than in controls [56]. In our data, however, BN participants exhibited reduced goal-directed motivation. This pattern of high punishment sensitivity and diminished reward pursuit may reflect a motivational imbalance that contributes to avoidant coping and emotion-driven binge-eating behaviors. For the BD-only group, reward sensitivity was comparable to or even lower than that of controls. The finding that the BAS-RR scores of the BD-only group were lower than those of the controls is consistent with the findings from two other studies [55, 57] indicating patients with depression may score lower on the BAS-RR than controls do. This reduced reward responsiveness is commonly observed in depressive states and may reflect the anhedonic features of bipolar depression, which, in turn, could reduce motivation and impair goal pursuit [55, 58].

Additionally, in the current study, negative urgency was comparably elevated in the BN–BD comorbid group and the BN-only group, and both groups scored higher than the BD-only group. Although negative urgency has been particularly predictive of binge-eating behavior, prior research has rarely explored its specific contribution to increased binge-eating symptoms in BN–BD comorbidity or in BD alone. Our findings support that negative urgency is particularly relevant to binge eating regardless of the presence of BN-BD comorbidity. With elevated negative urgency, patients with BD may also engage in binge eating in response to intense negative emotions, indicating that even without meeting full diagnostic criteria for BN, individuals with BD may still display emotional eating tendencies. This may explain their higher scores in binge eating (BITE) and body image concern (BSQ) compared to controls.

Based on the current findings, our study suggest that negative urgency has more specific relevance to binge eating psychopathology and attentional impulsivity may play a greater role in external-directed impulsive behaviors among individuals with BN. Future research should explore whether attentional impulsivity alone or in interaction with other variables including negative urgency, can better explain the increased co-occurrence of externally-directed impulsive behaviors in comorbid BN–BD compared to disease-only groups. In addition to using pharmacotherapy while considering BD comorbidity, our findings indicate the importance of targeting emotion regulation skills as well as behavioral control of binge eating and associated impulsive behaviors among individuals with BN. These findings further underscore the value of differentiating clinical features and impulsivity profiles associated with BN–BD comorbidity to guide more effective treatment planning in BN.

This study has several limitations. The limited sample size and the exclusive inclusion of female participants may limit the generalizability of our findings to more diverse populations. Furthermore, the current study’s cross-sectional design and reliance on self-report measures may have introduced bias, and therefore, future research should incorporate longitudinal designs and objective assessments, such as the Go/No-Go task, Stroop task, or Stop-Signal task. However, this study is the first to comprehensively compare emotional dysregulation and impulsivity across BN, BD, and comorbid BN–BD through multidimensional approaches. By categorizing participants into BN-only, BD-only, and BN–BD comorbid groups, the current study provides a nuanced perspective and contributes to an integrative understanding of the shared and distinct psychopathology underlying these conditions.

## Conclusion

Our findings suggest that individuals with BN and BD exhibit overlapping emotional dysregulation and impulsivity. Negative urgency and attentional impulsivity may have major contributions to binge eating and external impulsive behaviors, respectively, in individuals with comorbid BN–BD.

## Data Availability

The data supporting the findings of this study are available from the corresponding author upon reasonable request.
